# Supplementary material for: A single-cell resolved genotype-phenotype map using genome-wide genetic and environmental perturbations
Source: Nat Commun. 2025 Mar 18;16:2645. doi: 10.1038/s41467-025-57600-4 (PMC11920212; doi:10.1038/s41467-025-57600-4)
Supplement: Supplementary file 1 — Supplementary Information [file 41467_2025_57600_MOESM1_ESM.pdf]

Supplementary Information

A single-cell resolved genotype-phenotype map using genome-wide genetic and environmental perturbations

Mariona Nadal-Ribelles[1,2,*], Carme Solé[1,2], Anna Diez-Villanueva [2], Camille Stephan-Otto Attolini[2], Yaima Matas[1,2], Lars Steinmetz[3,4], Eulalia de Nadal[1,2,*] and Francesc Posas[1,2,*]

[1]Department of Medicine and Life Sciences, Universitat Pompeu Fabra. Barcelona, Spain

[2]Institute for Research in Biomedicine (IRB Barcelona), the Barcelona Institute of Science and Technology. Barcelona, Spain

[3]Department of Genetics, Stanford University, School of Medicine, California, USA.

[4]European Molecular Biology Laboratory, Heidelberg, Germany.

[*]Corresponding authors; mariona.nadal@irbbarcelona.org (MNR); eulalia.nadal@irbbarcelona.org (EdeN), francesc.posas@irbbarcelona.org (FP).

The file includes:

- Supplementary Figures 1 to 5.
- Supplementary Tables:
  - Supplementary Table 1: Plasmids generated in this study.
  - Supplementary Table 2: Yeast strains generated in this study.
- Supplementary References.

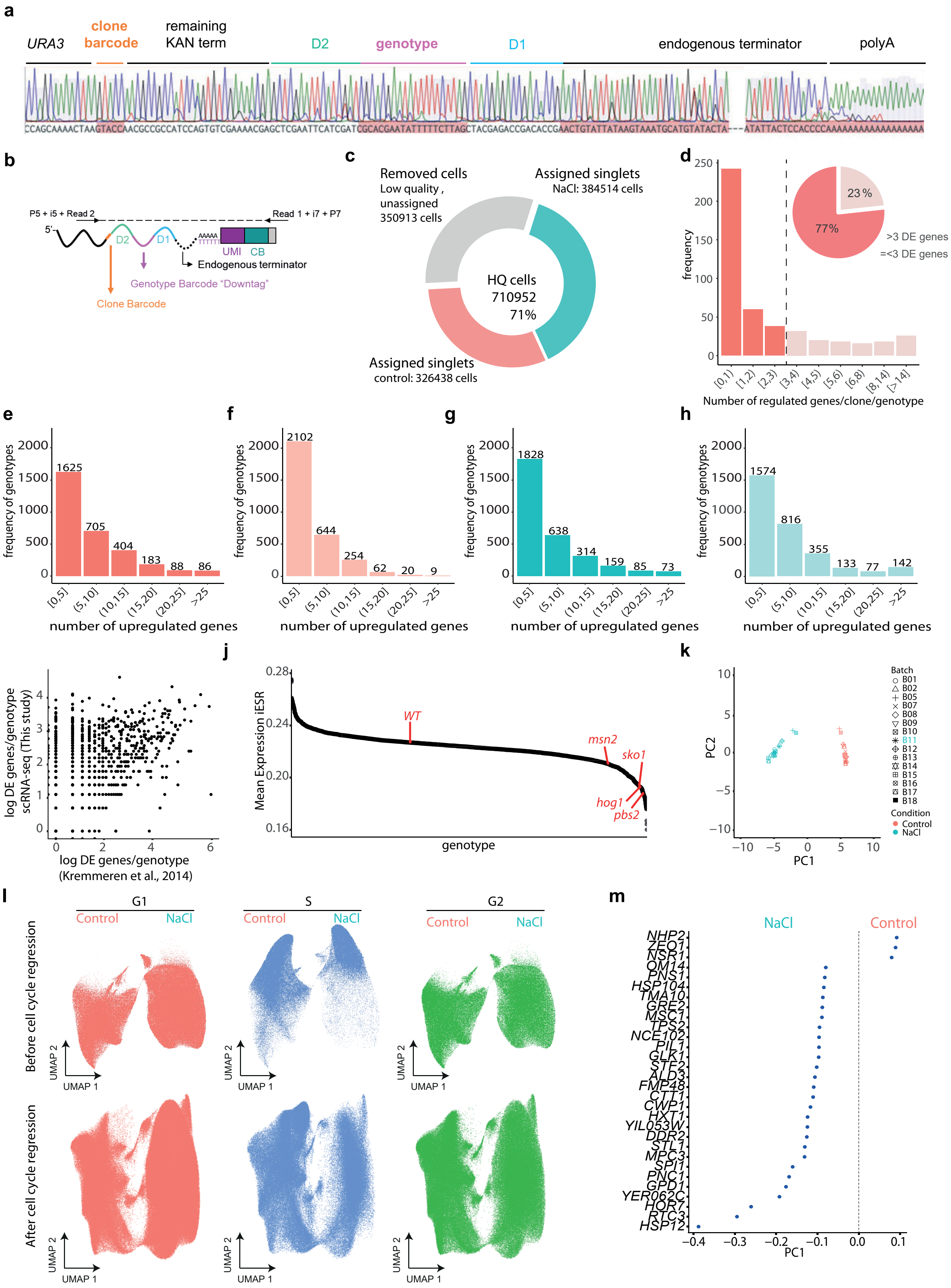
a
URA3
clone barcode
remaining KAN term
D2
genotype
D1
endogenous terminator
polyA
b
P5 + i5 + Read 2
Read 1 + i7 + P7
5'
D2
D1
UMI
CB
Endogenous terminator
Genotype Barcode "Downtag"
Clone Barcode
c
Removed cells
Low quality, unassigned
350913 cells
Assigned singlets
NaCl: 384514 cells
HQ cells
710952
71%
Assigned singlets
control: 326438 cells
d
frequency
23 %
77%
>3 DE genes
=<3 DE genes
Number of regulated genes/clone/genotype
e
frequency of genotypes
1625
705
404
183
88
86
number of upregulated genes
f
2102
644
254
62
20
9
number of upregulated genes
g
1828
638
314
159
85
73
number of upregulated genes
h
1574
816
355
133
77
142
number of upregulated genes
i
log DE genes/genotype scRNA-seq (This study)
log DE genes/genotype (Kremmeren et al., 2014)
j
Mean Expression iESR
WT
msn2
sko1
hog1
pbs2
genotype
k
PC2
PC1
Batch
B01
B02
B05
B07
B08
B09
B10
B11
B12
B13
B14
B15
B16
B17
B18
Condition
Control
NaCl
l
G1
S
G2
Control
NaCl
Before cell cycle regression
After cell cycle regression
UMAP 2
UMAP 1
m
NaCl
Control
NHP2
ZEO1
NSR1
OM14
PNS1
HSP104
TMA10
GRE2
MSC1
TPS2
NCE102
PIL1
GLK1
STF2
ALD3
FMP48
CTT1
CWP1
HXT1
YIL053W
DDR2
STL1
MPC3
SPI1
PNC1
GPD1
YER062C
HOR7
RTC3
HSP12
PC1

Supplementary Fig. 1: Redesign of the YKO collection with RNA-traceable deletions to enable genome-scale genetic and environmental perturbation screens.

a Schematic representation of the RNA-barcoded structure and a representative Sanger sequencing chromatogram. **b** Overview of the targeted amplification PCR strategy, the *URA3* transcript and the contained barcodes are shown in colors. Rectangle represents the anchoring oligo dT. Arrows indicate the location of primers and dotted line the amplification product. **c** Distribution of cells in the entire dataset after quality check (QC). Plot represents a total of 1.061.865 cells. Colored regions show removed cells (low quality cells or doublet cells, grey) or cells that pass the QC. The total number per each section and kept for further analysis are shown. **d** Histogram the classification of clones based on the number of differentially expressed (FC>2 *pvalue* <0.05). Only genotypes with at least 10 cells per clone. Pie chart on top, depicts the percentage of clones that express >3 differentially expressed genes (n=365) (see online Methods). Bars and pie chart are colored according to the number of differential expressed genes and dashed line indicates the threshold. **e-h** Histograms depicting the frequency of number of differentially expressed genes across genotypes in control (red bars) and stress (blue bars). Histograms shows bins of 5 genes and an overflow bin for >25 genes. Dark colored graphs represent the number of upregulated genes (FC>1 *pvalue* <0.05). Light graphs represent downregulated genes (FC>-1, *pvalue*<0.05). **i** Scatter plot showing the median (log) number of differentially expressed genes in control conditions for the intersecting mutants from bulk microarray profiling (Kemmeren et al., 2014) [1]. **j** Ranked mean iESR expression (y axis) across mutants (x axis) under stress condition. Wild type and reference mutants reported in the literature to display impaired induction of the response are highlighted in red. **k** Principal component analysis of the entire dataset. For each batch and condition the average value for PC1 and PC2 are represented. for each batch (shape) and colored by condition. Batch 11 contained only NaCl sample. **l** UMAP of the entire dataset colored by the indicated cell cycle phase. Cell cycle was determined using Seurat and canonical phase specific genes (see Methods). **m** Gene loadings of PC1 for Fig. 1j. Source data are provided as a Source Data file.

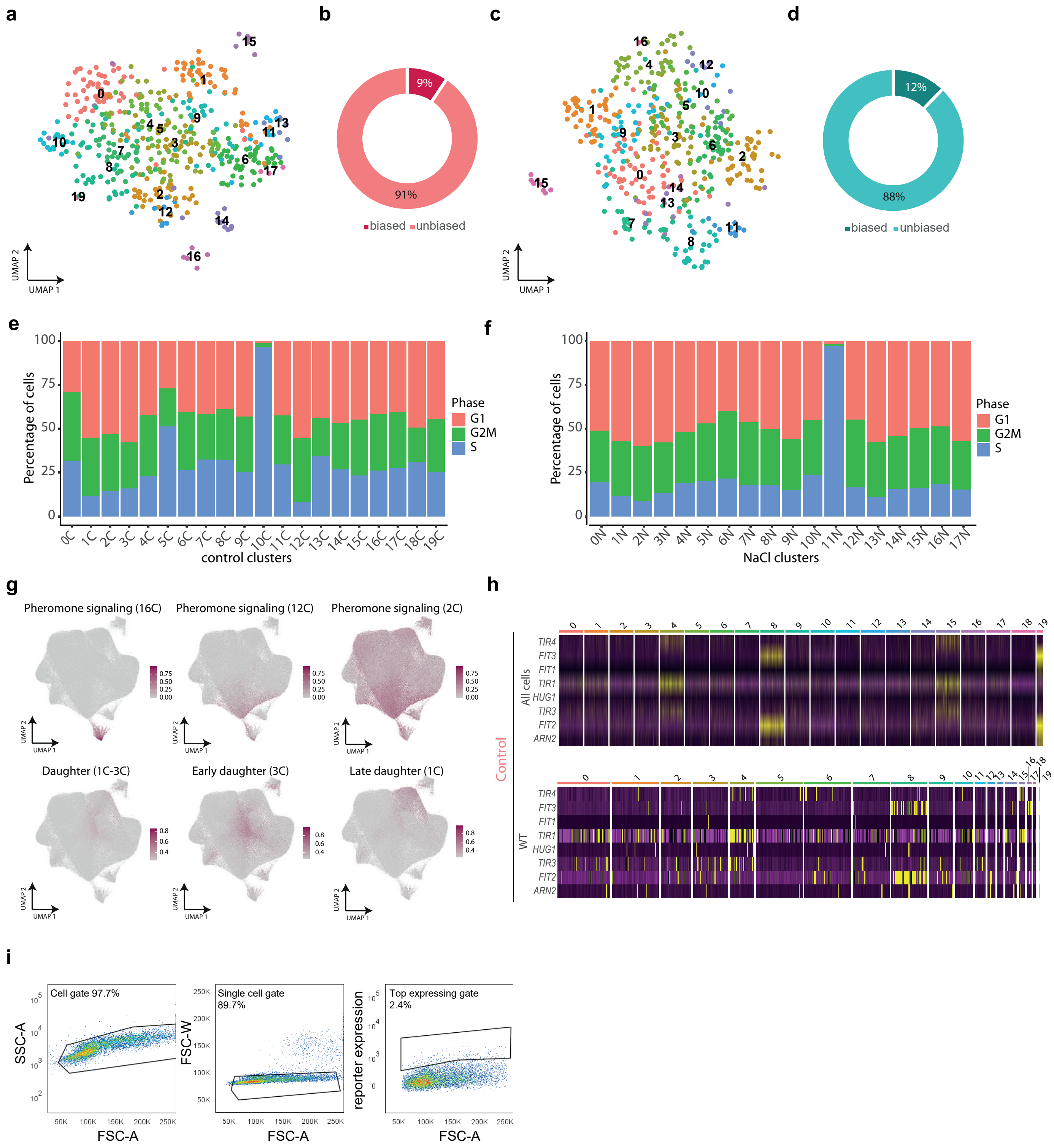

a
b
c
d
9%
91%
biased
unbiased
12%
88%
UMAP 1
UMAP 2
e
f
Percentage of cells
Phase
G1
G2M
S
control clusters
NaCl clusters
g
Pheromone signaling (16C)
Pheromone signaling (12C)
Pheromone signaling (2C)
Daughter (1C-3C)
Early daughter (3C)
Late daughter (1C)
h
All cells
WT
Control
TIR4
FIT3
FIT1
TIR1
HUG1
TIR3
FIT2
ARN2
i
Cell gate 97.7%
Single cell gate 89.7%
Top expressing gate 2.4%
SSC-A
FSC-W
reporter expression
FSC-A

Supplementary Fig. 2: Cells in a population arrange in heterogeneous gene expression states associated with biological function.

a UMAP of wild type cells extracted from the control dataset colored and labeled by cell state. **b** Percentage of mutants in control condition with unbiased cell distribution (light red) or biased distribution (dark red). **c** UMAP of wild type cells as in (A). **d** Percentage of mutants according to their distribution unbiased (light blue) or biased (dark blue). **e-f** Barplot represents the percentage of cells in each cell cycle phase for each cell state for control (e) or NaCl (f) dataset. **g** UMAP representing the expression signature defined by two genes of control clusters related to pheromone signaling (clusters 16C, 12C and 2C, upper panel). Projection of daughter cell state markers clusters (1C and 3C independently and combined). Darker color indicates higher expression. **h** Heatmap represents the expression of the aging signature for a downsampled subset of the entire control dataset (upper panel) and wild type cells only (lower panel). **i** Cells were gated sequentially using FSC-A/SSC-A, FSC-A/FSC-W (for single cell isolation), and reporter fluorescence (mCherry/iRFP) to isolate the top 2% expressing populations. For competition assays and expression analysis, cells were gated using FSC-A/SSC-A and analyzed for gene expression or population of interest (see Methods).

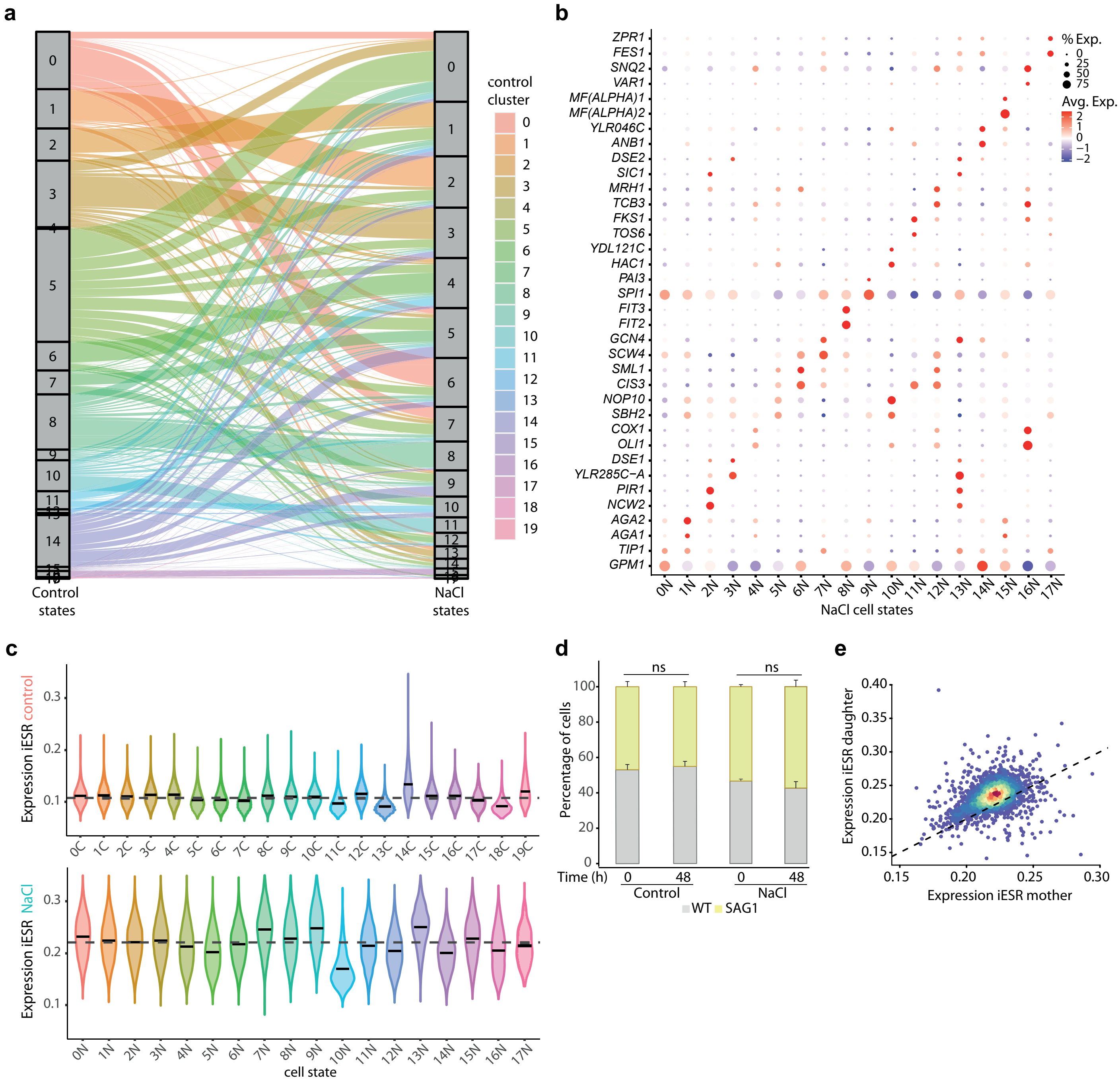
a
b
c
d
e
control cluster
Control states
NaCl states
% Exp.
Avg. Exp.
ZPR1
FES1
SNQ2
VAR1
MF(ALPHA)1
MF(ALPHA)2
YLR046C
ANB1
DSE2
SIC1
MRH1
TCB3
FKS1
TOS6
YDL121C
HAC1
PAI3
SPI1
FIT3
FIT2
GCN4
SCW4
SML1
CIS3
NOP10
SBH2
COX1
OLI1
DSE1
YLR285C−A
PIR1
NCW2
AGA2
AGA1
TIP1
GPM1
NaCl cell states
Expression iESR control
Expression iESR NaCl
cell state
Percentage of cells
ns
Time (h)
Control
NaCl
WT
SAG1
Expression iESR daughter
Expression iESR mother

Supplementary Fig. 3: Transcriptome mapping unveils core and responsive cell states that determine fitness.

a The alluvial plot represents percentage of corresponding cells pairs across the indicated clusters and conditions obtained from performing a label transfer. Clusters identified in the control condition (left) were used as a reference to predict cell type labels in the stress (right). The streams are colored based on the control clusters and indicate the connectivity across the indicated clusters (see Supplementary Data 5). **b** Expression of two representative cell state marker genes of the stress dataset. Dot size represent the percentage of expressing cells and are colored from high (red) to low (blue) expression. **c** Expression of the iESR signature for all cell states in each condition compared to the median of population (grey line). **d** Cells with the top 2% *SAG1* expression (p*SAG1*-UbiM-mCherry-t*SAG1*) were sorted and grown in combination with wild type cells (labelled with GFP) in rich media (control) or in the presence of stress (NaCl). Growth of the two populations was assessed at time 0 or after 48 hours by flow cytometry. Data represents mean and standard deviation of three independent experiments. Paired t-test comparing time 0h and 48h of each condition is shown on top (n=3). *p value* (control; 0,47 and NaCl 0.14). **e** Scatter plot shows the expression of the iESR signature (n=175 genes) per each genotype as a function of generation (mother cells, x axis and daughter cells y axis). Each point represents a genotype and points are colored by density (warmer colors indicate higher density). Source data are provided as a Source Data file.

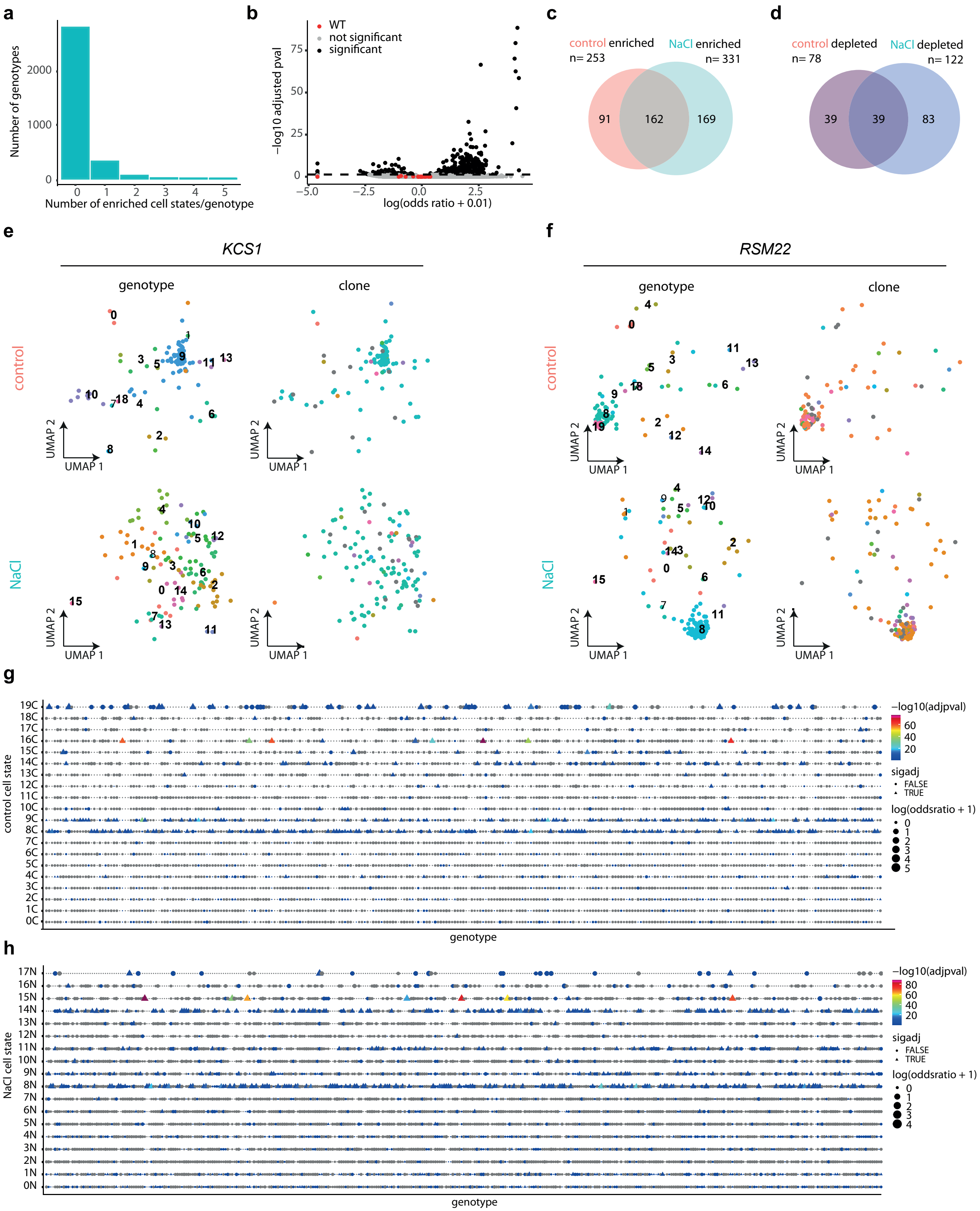
a
Number of genotypes
Number of enriched cell states/genotype
b
WT
not significant
significant
−log10 adjusted pval
log(odds ratio + 0.01)
c
control enriched
n= 253
NaCl enriched
n= 331
91
162
169
d
control depleted
n= 78
NaCl depleted
n= 122
39
39
83
e
KCS1
f
RSM22
genotype
clone
control
NaCl
UMAP 1
UMAP 2
g
control cell state
genotype
−log10(adjpval)
sigadj
FALSE
TRUE
log(oddsratio + 1)
h
NaCl cell state
genotype
−log10(adjpval)
sigadj
FALSE
TRUE
log(oddsratio + 1)

Supplementary Fig. 4: Single-cell genotype-to-phenotype analysis highlights regulators of yeast cell states.

a Distribution of the number of significant cell states per genotype in stress conditions. **b** Volcano plot shows the cell state enrichment of mutants (wild type is shown in red) in control conditions. Black line and dots show the threshold for statistical significance (two-sided Fisher test Benjamini-Hochberg adjusted *pvalue* <0.05). **c-d** Overlap between cell state enriched (c) or depleted (d) genotypes across conditions. **e-f** UMAP representation of genetically determined cell state for the indicated mutants across conditions (top/bottom panels). Points are colored and labeled based on the Seurat cluster (left panel) or based on their clone identity (grey points represent unassigned clones). **g-h** Phenomap shows the top500 genotypes with biased cell state enrichment (x axis) as a function of cell state (y axis) in control (g) and stress (h). Shape indicates statistical significance and are colors indicate the odds ratio value. Source data are provided as a Source Data file.

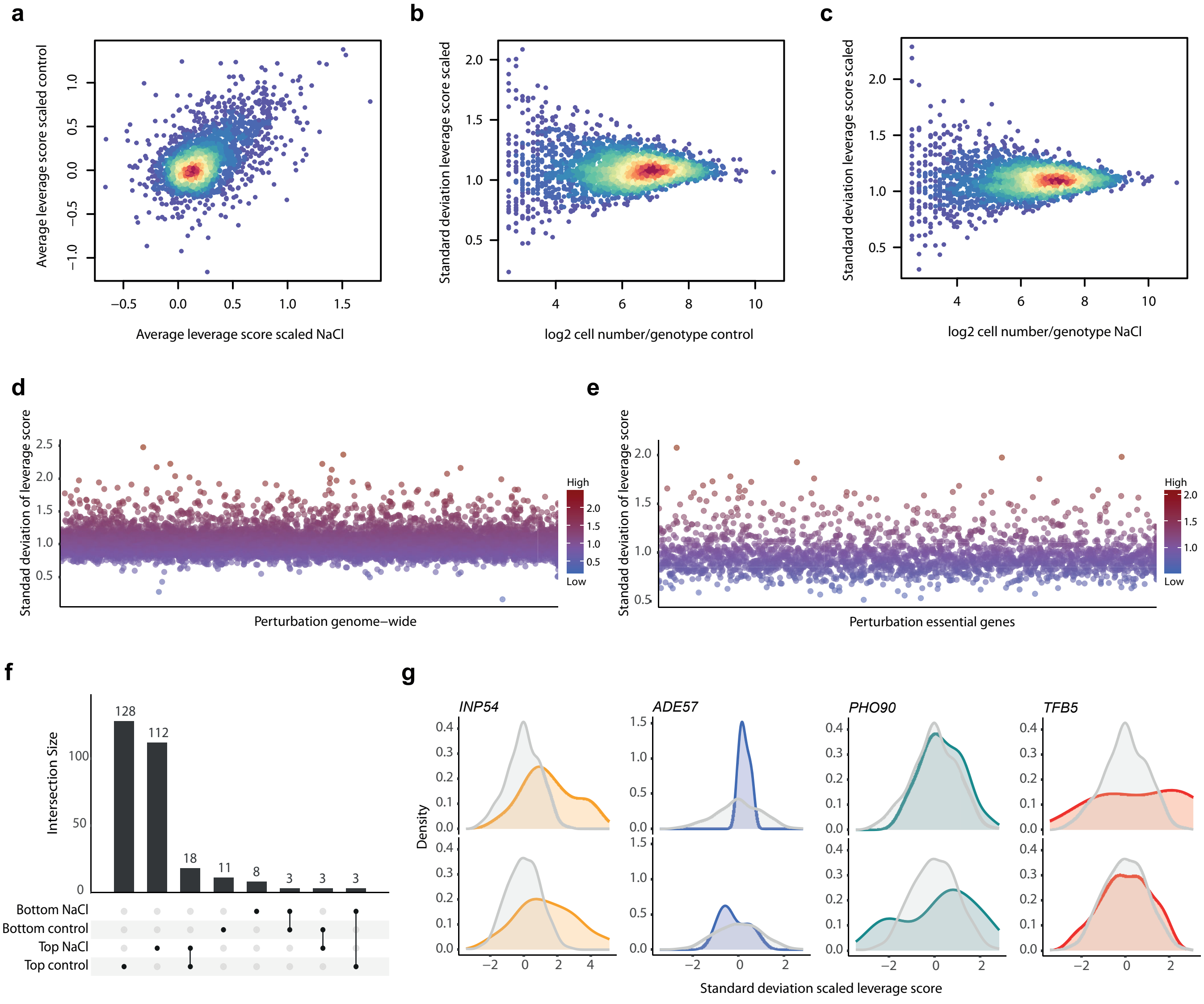

a
Average leverage score scaled control
Average leverage score scaled NaCl
b
Standard deviation leverage score scaled
log2 cell number/genotype control
c
Standard deviation leverage score scaled
log2 cell number/genotype NaCl
d
Standad deviation of leverage score
Perturbation genome−wide
High
Low
e
Standad deviation of leverage score
Perturbation essential genes
High
Low
f
Intersection Size
128
112
18
11
8
3
3
3
Bottom NaCl
Bottom control
Top NaCl
Top control
g
INP54
ADE57
PHO90
TFB5
Density
Standard deviation scaled leverage score

Supplementary Fig. 5: Different cellular functions drive transcriptional heterogeneity under control and stress conditions.

a Correlation of the average leverage score per each genotype (blue dots) in control (x axis) and stress (y axis) conditions. **b-c** Distribution of standard deviation of scaled leverage score (y axis) against the number of cells per genotype in control (b) and NaCl (c) datasets. Point density is shown in warmer colors (high) and colder colors (low). **d-e** Distribution of the standard deviation of scaled leverage score (y axis) in human Perturb-seq in K562 genome-scale (d) and essential gene screens (e) (Replogle et al., 2022)[2]. **f** Upset plot shows the overlap between negative and positive regulators identified in each condition. Bars are ordered in descending order in each indicated category and the total number is shown above. **g** Representative distributions of transcriptional heterogeneity (leverage score) patterns for the indicated mutants (colored distributions). The background filled distribution in grey is shown for the wild type strain in each condition. Source data are provided as a Source Data file.

Supplementary Table 1. List of plasmids used for experimental validation.

Plasmid num	Plasmid Name	Description	Source
pDC198	pYTK-Spect-*LEU2*_int-*HIS3* (pYTK147)	backbone for integrative plasmids	Canadaell et al.[3]
pNO82	pYTK-Nat-HO_int-Nat (pYTK168)	backbone for integrative plasmids	This study
pRP093	pYTK147-*pFIT3*-UbiM-mCherry 3b-*tFIT3*	Integrating plasmid carrying the *FIT3* reporter consisting of the promoter of *FIT3*, an N-terminal degradation signal fused to mCherry and terminator *FIT3*.	This study
pRP108	pYTK147-pHUG1 dBsaI-UbiM-mCherry 3b-tHUG1	Integrating plasmid carrying the *HUG1* reporter consisting of the promoter of HUG1, an N-terminal degradation signal fused to mCherry and terminator *HUG1*. dBsaI indicates the internal BsaI site was mutated to enable correct assembly.	This study
pRP128	pYTK168-pHSP12-UbiM-iRFP 3b-tHSP12	Integrating plasmid carrying the *HSP12* reporter consisting of the promoter of *HSP12*, an N-terminal degradation signal fused to iRFP and terminator *HSP12*.	This study
pDC448	pYTK144-TEF1i-YmukG1-tTDH1	Integrating plasmid carrying the *TEF1* reporter consisting of the promoter of TEF1, an N-terminal degradation signal fused to YmukG1 and terminator *TDH1*.	This study
pRP102	pYTK147-pSAG1-UbiM-mCherry 3b-tSAG1	Integrating plasmid carrying the *SAG1* reporter consisting of the promoter of *SAG1*, an N-terminal degradation signal fused to mCherry and terminator *SAG1*.	This study

Supplementary Table 2. List of strains generated in this study for experimental validation.

Strain name	Genotype	Source
yRP148	BY4741-pYTK147-pFIT3-UbiM-mCherry 3b- tFIT3:HIS	This study
yRP171	BY4741-pYTK147-pHUG1 dBsaI-UbiM-mCherry 3b- tHUG1:HIS	This study
yRP178	BY4741-pYTK144-TEF1i-YmukG1-tTDH1:LEU	This study
yRP193	BY4741-pYTK168-pHSP12-UbiM-IRFP 3b-tHSP12: NAT	This study
yRP465	BY4741-pYTK147-pSAG1-UbiM-mCherry 3b- tSAG1:HIS	This study
yRP211	MRP7::URA3-pYTK147-pFIT3-UbiM-mCherry-tFIT3:HIS	This study
yRP225	MSS116::URA3-pYTK147-pFIT3-UbiM-mCherry-tFIT3:HIS	This study
yRP231	RIM8::URA3 (4_G)-pYTK147-pFIT3-UbiM-mCherry-tFIT3:HIS	This study
yRP241	SNF7::URA3-pYTK147-pFIT3-UbiM-mCherry-tFIT3:HIS	This study
yRP264	YMN479-pYTK147-pFIT3-UbiM-mCherry-tFIT3 (WT)	This study
NA	SAS4::URA3	This study
NA	SAS5::URA3	This study
NA	YPL216W::URA3	This study

Supplementary References

1. Kemmeren, P. *et al.* Large-Scale Genetic Perturbations Reveal Regulatory Networks and an Abundance of Gene-Specific Repressors. *Cell* **157**, 740–752 (2014).

2. Replogle, J. M. *et al.* Mapping information-rich genotype-phenotype landscapes with genome-scale Perturb-seq. *Cell* **185**, 2559-2575.e28 (2022).

3. Canadell, D. *et al.* Implementing re-configurable biological computation with distributed multicellular consortia. *Nucleic Acids Res* **1**, 1–18 (2022).
