# Supplementary material for: A single-cell resolved genotype-phenotype map using genome-wide genetic and environmental perturbations
Source: Nat Commun. 2025 Mar 18;16:2645. doi: 10.1038/s41467-025-57600-4 (PMC11920212; doi:10.1038/s41467-025-57600-4)
Supplement: Supplementary file 2 — Description of Additional Supplementary Files [file 41467_2025_57600_MOESM2_ESM.pdf]

Description of Additional Supplementary Files

File Name: Supplementary Data 1
Description: Excel file with the distance to expected chromosome location (annotated STOP codon) of each genotype.
File Name: Supplementary Data 2
Description: Excel file with the marker for each cell state in control. Each tab also includes the Gene Enrichment Table for the upregulated genes.
File Name: Supplementary Data 3
Description: Excel file with the marker for each cell state in NaCl. Each tab also includes the Gene Enrichment Table for the upregulated genes.
File Name: Supplementary Data 4
Description: Excel file containing the percentage of cells in each cell cycle phase for each genotype under control and NaCl.
File Name: Supplementary Data 5
Description: Excel file contains the frequency of cells in each predicted cell state for both control and NaCl.
File Name: Supplementary Data 6
Description: Excel file with the cell state enrichment of each genotype in control.
File Name: Supplementary Data 7
Description: Excel file with the cell state enrichment per genotype in NaCl.
File Name: Supplementary Data 8
Description: Excel file containing the leverage score metrics per each genotype in control and NaCl.
File Name: Supplementary Data 9
Description: Excel file containing the gene names used to score gene signatures.
