# Supplementary material for: A single-cell resolved genotype-phenotype map using genome-wide genetic and environmental perturbations
Source: Nat Commun. 2025 Mar 18;16:2645. doi: 10.1038/s41467-025-57600-4 (PMC11920212; doi:10.1038/s41467-025-57600-4)
Supplement: Supplementary file 12 — Reporting Summary [file 41467_2025_57600_MOESM12_ESM.pdf]

nature portfolio

Corresponding author(s): Francesc Posas
Eulàlia de Nadal
Mariona Nadal-Ribelles

Last updated by author(s): Jan 30, 2025

Reporting Summary

Nature Portfolio wishes to improve the reproducibility of the work that we publish. This form provides structure for consistency and transparency in reporting. For further information on Nature Portfolio policies, see our Editorial Policies and the Editorial Policy Checklist.

Statistics

For all statistical analyses, confirm that the following items are present in the figure legend, table legend, main text, or Methods section.

n/a	Confirmed	
☐	☒	The exact sample size (*n*) for each experimental group/condition, given as a discrete number and unit of measurement
☐	☒	A statement on whether measurements were taken from distinct samples or whether the same sample was measured repeatedly
☐	☒	The statistical test(s) used AND whether they are one- or two-sided<br>*Only common tests should be described solely by name; describe more complex techniques in the Methods section.*
☒	☐	A description of all covariates tested
☐	☒	A description of any assumptions or corrections, such as tests of normality and adjustment for multiple comparisons
☐	☒	A full description of the statistical parameters including central tendency (e.g. means) or other basic estimates (e.g. regression coefficient) AND variation (e.g. standard deviation) or associated estimates of uncertainty (e.g. confidence intervals)
☐	☒	For null hypothesis testing, the test statistic (e.g. *F*, *t*, *r*) with confidence intervals, effect sizes, degrees of freedom and *P* value noted<br>*Give P values as exact values whenever suitable.*
☒	☐	For Bayesian analysis, information on the choice of priors and Markov chain Monte Carlo settings
☒	☐	For hierarchical and complex designs, identification of the appropriate level for tests and full reporting of outcomes
☐	☒	Estimates of effect sizes (e.g. Cohen's *d*, Pearson's *r*), indicating how they were calculated

Our web collection on statistics for biologists contains articles on many of the points above.

Software and code

Policy information about availability of computer code

Data collection	Yeast Perturb-seq experiments were performed using the Yeast Singleron HD ChIP and using their matrix system. The generated libraries of transcriptome and targeted amplification were sequenced as FASTQ files were sued as an input for pre-processing. Transcriptome libraries were processed using the open source CeleScope pipeline developed by Singleron Biotechnologies. The targeted amplification was performed from the same full length cDNA libraries used for transcriptome library generation using custom primers (see Methods section). Targeted amplication was analyzed using custom code.
Data analysis	For the data analysis. The open source CeleScope was used using default parameters. The output folders for all samples (transcriptome libraries) were used as an input to generate Seurat objects and R Studio and published R packages we used. For the targeted amplification a custom code was used. The raw data has been subitted to Array Express, the code to reprodice the figures has been uploaded to Zenodo (10.5281/zenodo.14062629) and fully processed Seurat objects of both the combined dataset and individual datasets have been also uploaded to the same Zenodo reporsitory (10.5281/zenodo.14062629). Direct links or accession number are provided within the manuscript.

For manuscripts utilizing custom algorithms or software that are central to the research but not yet described in published literature, software must be made available to editors and reviewers. We strongly encourage code deposition in a community repository (e.g. GitHub). See the Nature Portfolio guidelines for submitting code & software for further information.

Data

Policy information about availability of data

All manuscripts must include a data availability statement. This statement should provide the following information, where applicable:

- Accession codes, unique identifiers, or web links for publicly available datasets
- A description of any restrictions on data availability
- For clinical datasets or third party data, please ensure that the statement adheres to our policy

The raw sequencing data for transcriptome and targeted amplification generate in this study are available in the Array Express database under accession code (E-MTAB-14004). Fully processed Seurat objects containing the individual or combined datasets are available through Zenodo (DOI: 10.5281/zenodo.14062629). Other data used in this manuscript: yeast fitness data across stressor panel were obtained from Supplementary Table 1 (Mutant Fitness Conditions) from https://pubmed.ncbi.nlm.nih.gov/33958448/, and leverage score from human Perturb-seq were obtained from Table S2 that contains Perturb-seq pseudobulk summary statistics from https://pubmed.ncbi.nlm.nih.gov/35688146/. Source data are provided with this paper.

Zenodo:
10.5281/zenodo.14062629

Research involving human participants, their data, or biological material

Policy information about studies with human participants or human data. See also policy information about sex, gender (identity/presentation), and sexual orientation and race, ethnicity and racism.

Reporting on sex and gender	does not apply
Reporting on race, ethnicity, or other socially relevant groupings	does not apply
Population characteristics	does not apply
Recruitment	does not apply
Ethics oversight	does not apply

Note that full information on the approval of the study protocol must also be provided in the manuscript.

Field-specific reporting

Please select the one below that is the best fit for your research. If you are not sure, read the appropriate sections before making your selection.

☒ Life sciences ☐ Behavioural & social sciences ☐ Ecological, evolutionary & environmental sciences

For a reference copy of the document with all sections, see nature.com/documents/nr-reporting-summary-flat.pdf

Life sciences study design

All studies must disclose on these points even when the disclosure is negative.

Sample size	No statistical methods were used to calculate sample size. For scRNA-seq
Data exclusions	We initially profiled a total of 1.061.865 cells. The expression matrices were generated with Celesope and used to remove low quality cells (-/+ 2 standard deviation of mean genes, >10% mitochondrial reads) and doublets. Cells for which a genotype could not be assigned were also removed from the analysis.
Replication	Experimental validations of the scRNA-seq dataset derive from three independent biological replicates. For the scRNA-seq dataset one replicate per condition.
Randomization	For assessment of aged phenotype such as scar counting and mitochondrial morphology, were counted randomly. As well for FACS analysis

Randomization	the entire single population was used to define either population abundance or gene expression. For the rest of the experiments not mentioned here randomization was not important.
Blinding	Generation of scRNA-seq libraries after cell partition including sequencing and data preprocessing (alignment and genotype calling) were done blindly.

Reporting for specific materials, systems and methods

We require information from authors about some types of materials, experimental systems and methods used in many studies. Here, indicate whether each material, system or method listed is relevant to your study. If you are not sure if a list item applies to your research, read the appropriate section before selecting a response.

Materials & experimental systems

n/a	Involved in the study
☐	☒ Antibodies
☒	☐ Eukaryotic cell lines
☒	☐ Palaeontology and archaeology
☐	☒ Animals and other organisms
☒	☐ Clinical data
☒	☐ Dual use research of concern
☒	☐ Plants

Methods

n/a	Involved in the study
☒	☐ ChIP-seq
☐	☒ Flow cytometry
☒	☐ MRI-based neuroimaging

Antibodies

Antibodies used	Lysine acetylation (Cell Signaling, 9441S) . 0.5 ug antibody per sample against Lysine acetylation (Cell Signaling, 9441S) .<br>25 µ rabbit Dynabeads® M-280 Sheep Anti-Rabbit IgG (Life Technologies, 11204D)
Validation	The information related to this antibody can be found here: https://www.cellsignal.com/products/primary-antibodies/acetylated-lysine-antibody/9441.<br>This antibody is suitable for chromatin immunoprecipitation experiments and is reactive with S. cerevisiae as stated by the vendor.

Animals and other research organisms

Policy information about studies involving animals; ARRIVE guidelines recommended for reporting animal research, and Sex and Gender in Research

Laboratory animals	does not apply
Wild animals	does not apply
Reporting on sex	does not apply
Field-collected samples	does not apply
Ethics oversight	does not apply

Note that full information on the approval of the study protocol must also be provided in the manuscript.

Plants

Seed stocks	does not apply
Novel plant genotypes	does not apply
Authentication	does not apply

Flow Cytometry

Plots

Confirm that:

☒ The axis labels state the marker and fluorochrome used (e.g. CD4-FITC).

☒ The axis scales are clearly visible. Include numbers along axes only for bottom left plot of group (a 'group' is an analysis of identical markers).

☒ All plots are contour plots with outliers or pseudocolor plots.

☒ A numerical value for number of cells or percentage (with statistics) is provided.

Methodology

Sample preparation	Samples analyzed by Flow cytometry were analyzed directly from the growth media. For expression of reporters cells were sorted in expoentnial phase. For competition experiments cells were fixed with formaldehyde in their media which from which they were anayzed
Instrument	For analysis: Cytek® Aurora (4-laser and 64 Fluorescence Emission Detection Channels)<br>For sorting: BD FACSAria III Cell Sorter
Software	Cytometry data were analyzed using FlowJo™ Software (BD Life Sciences).
Cell population abundance	For reporter analysis a total of 10,000 cells were used to determine expression distribution.<br>For competition assays at time 0 a total of 1000 cells were used to determine initial cell ratios.
Gating strategy	The gating strategy follows standard procedures where cell population is selected through gating cell size and complexity (FSC and SSC) using the FlowJo. The degree of expression reported is always represented from this gate.

☒ Tick this box to confirm that a figure exemplifying the gating strategy is provided in the Supplementary Information.
